# Development and Evaluation of Rehabilitation Service Areas for the United States

**DOI:** 10.1186/s12913-023-09184-2

**Published:** 2023-03-01

**Authors:** Timothy A. Reistetter, Julianna M. Dean, Allen M. Haas, John D. Prochaska, Daniel C. Jupiter, Karl Eschbach, Yong-Fang Kuo

**Affiliations:** 1 University of Texas Health Science Center at San Antonio, 7703 Floyd Curl Dr, San Antonio, TX 78229 USA; 2 University of Houston-Clear Lake, 2700 Bay Area Blvd, Houston, TX 77058 USA; 3 The University of Texas MD Anderson Cancer Center, 1515 Holcombe Boulevard, Houston, TX 77030 USA; 4 The University of Texas Medical Branch, 301 University Blvd, Galveston, TX 77555 USA

**Keywords:** Post-acute care, Small-area variation, Geographic variation, Medicare, Ward’s clustering method

## Abstract

**Background:**

Geographic areas have been developed for many healthcare sectors including acute and primary care. These areas aid in understanding health care supply, use, and outcomes. However, little attention has been given to developing similar geographic tools for understanding rehabilitation in post-acute care. The purpose of this study was to develop and characterize post-acute care Rehabilitation Service Areas (RSAs) in the United States (US) that reflect rehabilitation use by Medicare beneficiaries.

**Methods:**

A patient origin study was conducted to cluster beneficiary ZIP (Zone Improvement Plan) code tabulation areas (ZCTAs) with providers who service those areas using Ward’s clustering method. We used US national Medicare claims data for 2013 to 2015 for beneficiaries discharged from an acute care hospital to an inpatient rehabilitation facility (IRF), skilled nursing facility (SNF), long-term care hospital (LTCH), or home health agency (HHA). Medicare is a US health insurance program primarily for older adults. The study population included patient records across all diagnostic groups. We used IRF, SNF, LTCH and HHA services to create the RSAs. We used 2013 and 2014 data (*n* = 2,730,366) to develop the RSAs and 2015 data (*n* = 1,118,936) to evaluate stability. We described the RSAs by provider type availability, population, and traveling patterns among beneficiaries.

**Results:**

The method resulted in 1,711 discrete RSAs. 38.7% of these RSAs had IRFs, 16.1% had LTCHs, and 99.7% had SNFs. The number of RSAs varied across states; some had fewer than 10 while others had greater than 70. Overall, 21.9% of beneficiaries traveled from the RSA where they resided to another RSA for care.

**Conclusions:**

Rehabilitation Service Areas are a new tool for the measurement and understanding of post-acute care utilization, resources, quality, and outcomes. These areas provide policy makers, researchers, and administrators with small-area boundaries to assess access, supply, demand, and understanding of financing to improve practice and policy for post-acute care in the US.

## Background

Regional differences in health care and outcomes have been studied for over forty years in the United States (US) [1]. Although regional differences are attributable to patient-level clinical factors, a substantial amount of variation is due to issues beyond the individual’s health status or other individual-level attributes. Some examples include the availability of or access to healthcare services, practice patterns, and geographic and environmental factors [2–7].{Institute of Medicine (U.S.). Committee on Geographic Variation in Health Care Spending and Promotion of High-Value Care, 2013, Variation in health care spending: target decision making`, not geography}.

Post-acute care (PAC) is no different than acute care or primary care in terms of the presence of regional variation [8–11]. Rehabilitation researchers have found geographic differences in PAC length of stay, utilization, and community discharge [12–16]. A critical step in understanding, addressing, and ultimately minimizing geographic differences in PAC outcomes is related to optimizing the geospatial area used to characterize geographic differences in PAC [17]. The most prominent group for developing and documenting geographic areas and subsequent variation is the Dartmouth Atlas Group [18]. They developed and manage geographic boundaries for acute hospital-based care as well as primary care. The most notable of these are Hospital Service Areas, Hospital Referral Regions, and Primary Care Service Areas [2, 19]. Other areas used to study geographic variation include administrative systems characterized for non-healthcare purposes like counties, states, ZIP (Zone Improvement Plan) code tabulation areas (ZCTAs), and census tracks.

Small-area variation, differences in healthcare use and outcomes across localized geographic areas, is not explained by patient-level clinical characteristics. Small-area variation techniques capture relatively localized geographic spaces through aggregation of ZIP codes where a set of healthcare providers dominate the delivery of services for individuals living in each geographic area [19]. This approach is commonly referred to as the “plurality rule”. With the plurality rule, areas are assigned to the provider who serves most of the individuals in that area. Although previous boundary systems for hospitals, primary care, and other service areas each used the plurality approach, these tools were developed for uniquely different purposes. For example, Primary Care Service Areas were designed to characterize primary care while Hospital Service Areas and Hospital Referral Regions were developed to capture secondary and tertiary care provided by acute care hospitals. Notably missing are geographic tools for examining variation in rehabilitation which has led rehabilitation researchers and policy makers to use service area tools designed for other areas of healthcare [20–22].

However, the use of the other service area tools for PAC research may be problematic. Further, PAC rehabilitation poses some unique challenges to developing and evaluating service areas using the plurality rule. The primary challenge is that rehabilitation consists of a group of different provider types [17]. Unlike primary care and hospital care, each type of rehabilitation provider has a unique payment structure and service delivery model. The four most common rehabilitation services include 1) inpatient rehabilitation facilities (IRF), 2) skilled nursing facilities (SNF), 3) long-term care hospitals (LTCH), and 4) home health agencies (HHA). Although patients with the same diagnosis may be treated in each of these settings, the treatments and rules governing these treatments vary [23].

Inpatient rehabilitation is for complex rehabilitation patients who are required to tolerate three hours of rehabilitative therapy per day from a combination of physical, occupational, and speech therapy providers. Further complicating the IRF setting is the sixty percent rule which requires that 60% of patients have one of 13 diagnoses over the course of the year. These diagnoses include 1) stroke, 2) spinal cord injury, 3) congenital deformity, 4) amputation, 5) major multiple trauma, 6) hip fracture, 7) brain injury, 8) certain neurological conditions (e.g., multiple sclerosis, Parkinson’s disease), 9) burns, 10–12) three arthritis conditions for which appropriate, aggressive, and sustained outpatient therapy has failed, and 13) hip or knee replacement when it is bilateral, when the patient’s body mass index is greater than or equal to 50, or when the patient is age 85 or older [24].

Skilled nursing rehabilitation is less intensive and does not have a three-hour treatment rule. Also, SNFs are not required to treat specific patient groups. Rather, the patient need only demonstrate rehabilitative potential [25].

Long-term care hospitals are specialty hospitals for critically ill patients who are stable, yet they require extensive support and are expected to take a longer period to rehabilitate. One example and diagnostic group seen in an LTCH setting are those with respiratory failure requiring ventilator support which represents the largest group of records nationally at 11% [26].

In contrast, home health agencies provide the least intensive level of care and often represent patients discharged directly home following an index event [27]. Those receiving home health services must meet homebound status requirements and are eligible to receive nursing and rehabilitation for an episode of care which is defined as 60 visits [27].

In addition to differences in intensity, admission practices, and treatment provided, each of these provider types has different staffing pattern requirements [28–30]. Furthermore, healthcare types are governed nationally and with state-specific statements of need requirements which impact opening and operating procedures [31–33]. These factors, unique to PAC services, influence utilization patterns [34]. Current geographic boundaries that use the plurality rule were not created with these factors in mind. Therefore, boundaries that incorporate all PAC types and utilization patterns are needed and may facilitate the examination of variation in PAC in the US.

In this study, we developed Rehabilitation Service Areas (RSAs) which are geographic areas for PAC utilization in the US. The purpose of this study was to define RSAs, detail the methodological approach to RSA construction, and determine RSA validity across the US. We discuss limitations of the method and future uses of the RSA boundaries. We propose RSAs as a solution to assess geographic variation in PAC more effectively than current geographic areas in use.

## Methods

### Aim, Design, and Setting

The aim of this study was to develop and characterize PAC RSAs in the US that reflect rehabilitation use by Medicare beneficiaries. We employed a patient origin study to cluster beneficiary ZCTAs with providers who service those areas using Ward’s clustering method [35, 36].

We used data from the Centers for Medicare and Medicaid Services (CMS). Medicare is a US health insurance program primarily for individuals 65 and older [37]. Medicare also covers individuals with disabilities who are under 65 years of age [37, 38]. However, use patterns differ for Medicare beneficiaries enrolled due to disability [38]. In this study, we used 100% CMS files for 2013 through 2015 from the Medicare Provider Analysis and Review file (MedPAR) [39], the Provider of Service (POS) file [40], the Medicare Beneficiary Summary file [39], and the Home Health Claims file [39]. The MedPAR file contains IRF, SNF, and LTCH records that detail information such as admission and discharge dates and facility identification numbers. The POS file is publicly available and contains provider details such as unique provider number and location. The Medicare Beneficiary Summary file includes beneficiary demographics. Finally, the Home Health claims file contains characteristics of home health visits such as skilled care and therapy services. Our sample included patient records from Medicare beneficiaries’ first acute discharges between January 01, 2013, to October 01, 2015. These data represented two samples. We used the 2013 and 2014 data for developing RSAs and 2015 data to evaluate the stability of the RSAs developed from the 2013 and 2014 data. Our initial sample for 2013 and 2014 was 12,685,681 acute care records. We removed patients younger than 66 years at discharge from acute care (*n* = 2,514,757). Although there are younger individuals who use Medicare due to disability, their PAC use patterns differ [37, 38]. Therefore, they were removed. We also excluded those without continuous fee-for-service Medicare coverage (*n* = 2,988,210), and those with discrepant ZCTA data (*n *= 761). To characterize PAC rehabilitation, we excluded those who had a long-term care claim within 90 days of acute discharge (*n* = 101,116), and those patients not admitted to an IRF, SNF, LTCH, or HHA based on provider number from the POS file (*n* = 4,298,507). Finally, we excluded those living outside the US (*n* = 9,795) and patients from ZCTAs with 10 or fewer patients (*n* = 42,169). This yielded a final cohort of 2,730,366 beneficiary records for the 2013–2014 data used to build RSAs. For the 2015 data used to test stability of the RSAs, the exclusion criteria were the same with similar percentages of records excluded at each step of the cohort selection. This resulted in a 2015 cohort of 1,118,936 records. The authors obtained a Data Use Agreement with CMS. This study was approved by The University of Texas Medical Branch Institutional Review Board.

### Basic Explanation of Ward’s Clustering Method

**Fig. 1:**
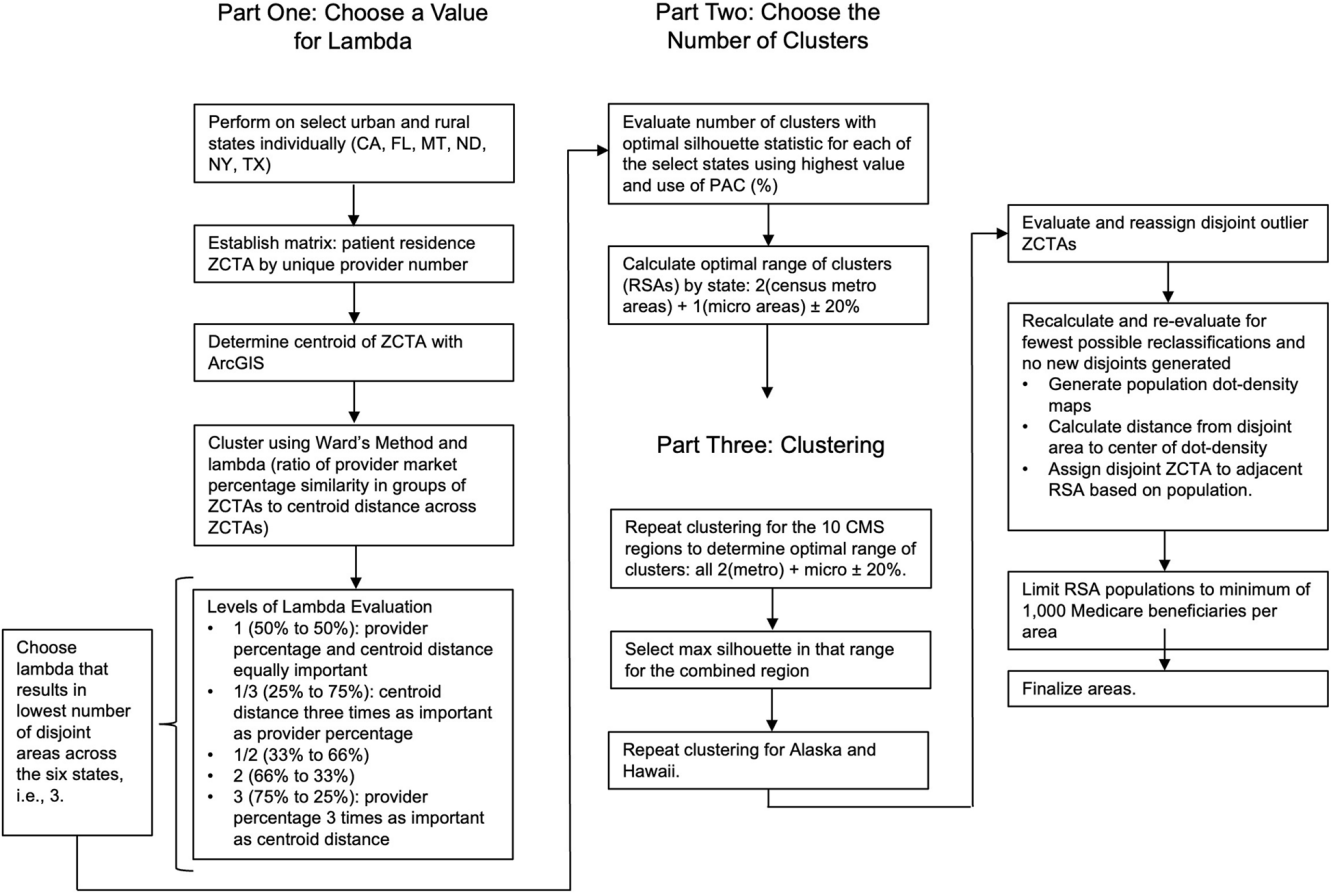
Flowchart of the study methods for creating Rehabilitation Service Areas including steps of Ward’s clustering method. “States” refers to US states, *CA* California, *FL* Florida, *MT* Montana, *ND* North Dakota, *NY* New York, *TX* Texas, *ZCTA* ZIP [Zone Improvement Plan] Code Tabulation Area, *PAC* Post-acute care, *RSA* Rehabilitation Service Area, *CMS* Centers for Medicare and Medicaid Services

Figure 1 is a flowchart of the study’s methods and consists of three main parts, each of which are explained below. Throughout, we used Ward’s clustering method to create the RSAs from ZCTAs [35, 36]. The clustering method begins by partitioning each element into its own cluster. Then, based on a provider distance matrix, it combines the two clusters that are the most similar. This process is repeated until all clusters are combined, creating the Ward’s clustering for each number of clusters between the number of elements and one.

To create the distance matrix necessary for use with Ward’s clustering method, a combination of provider percentage for each ZCTA and geographic distance between ZCTAs was used. To determine the provider percentage for each ZCTA, the proportion of beneficiaries using each IRF, SNF, LTCH, and HHA was determined. Then, a distance matrix was created based on the differences in provider percentage patterns between ZCTAs. For the geographic distance component, physical distance between ZCTA centroids was used. The two components were then combined, with a weighting ratio Lambda. Lambda is the relative weight of the provider percentage and geographic data. Therefore, a Lambda of 2 would indicate the provider percentage is twice as important as the geographic data (i.e., 66% provider percentage, 33% geographic data).

### Part One: Choose a Value for Lambda

In part one, we conducted an exploratory analysis to determine an appropriate value for Lambda. We selected six states for the analysis. California, Florida, Montana, North Dakota, New York, and Texas were chosen for their variety in terms of population density. We then ran our clustering algorithm with several values of lambda: 1, 1/3, 1/2, 2, and 3. Based on the resultant clusters, we chose Lambda = 3, as it yielded the fewest disjoint clusters.

### Part Two: Choose the Number of Clusters

Because Ward’s clustering method does not choose the number of clusters, it is necessary to establish a criterion for doing so. For our study, we used the silhouette statistic to evaluate how well a certain clustering performed relative to other clusterings. The silhouette is a clustering statistic that measures how similar elements are within a cluster compared to other clusters [41]. Higher values indicate that elements match those in the cluster and are different than neighboring clusters. For our study, we considered the mean silhouette for all its ZCTAs for each clustering, and we considered a clustering to be better if it had a higher mean silhouette.

When clustering, we found that for our data, the silhouette initially rose rapidly with the number of clusters and then flattened into a long plateau. This shape structure proved problematic, as maximizing the silhouette statistic produced too many clusters to be useful, whereas choosing the inflection point (the point where the silhouette turned from its rapid rise into the plateau) produced too few clusters for our purposes. To account for this, we chose to maximize the silhouette statistic within a range of values. Based on exploratory work done on Texas PAC [17], we used two times the metropolitan areas plus one time the number of micropolitan areas per state, plus or minus 20%. For example, in Texas, there are 25 metro areas and 46 micro areas. This results in 96 areas plus or minus 20%, which yields a range of 77–115 areas for Texas. Metropolitan areas are defined by Census Core Based Statistical Areas as largely populated urban areas with a minimum of 50,000 residents, while micropolitan areas are community areas with a population of 10,000 with a high degree of social and economic integration.

### Part Three: Clustering

Clustering the entire US at once quickly became infeasible, as combining the large variety of rural areas and urban areas resulted in many singleton or disjoint clusters. Given the potential for traveling across state boundaries for services in IRFs, SNFs, and LTCHs, and the infeasibility of clustering the entire country, we elected to cluster the continental US using the ten CMS regions, and then we separately clustered Hawaii and Alaska. CMS divides the US into 10 administrative areas. Each region includes multiple states which have been used to evaluate rehabilitation outcomes nationally [42]. The following states are represented in the 10 CMS regions: 1) Connecticut, Maine, Massachusetts, New Hampshire, Rhode Island, Vermont, 2) New Jersey, New York, Puerto Rico, Virgin Islands, 3) Delaware, District of Columbia, Maryland, Pennsylvania, Virginia, West Virginia, 4) Alabama, Florida, Georgia, Kentucky, Mississippi, North Carolina, South Carolina, Tennessee, 5) Illinois, Indiana, Michigan, Minnesota, Ohio, Wisconsin, 6) Arkansas, Louisiana, New Mexico, Oklahoma, Texas, 7) Iowa, Kansas, Missouri, Nebraska, 8) Colorado, Montana, North Dakota, South Dakota, Utah, Wyoming, 9) American Samoa, Arizona, California, Guam, Hawaii, Nevada, and 10) Alaska, Idaho, Oregon, and Washington [42, 43]. Therefore, we applied the formula of two times the number of metro areas plus the number of micro areas in that region plus or minus 20%, thus allowing areas to cross state borders for IRF, SNF, and LTCH services. As a sensitivity analysis, we combined CMS regions 1 & 2 as well as CMS regions 2, 3, & 5 and found very few inter-region clusters.

**Fig. 2:**
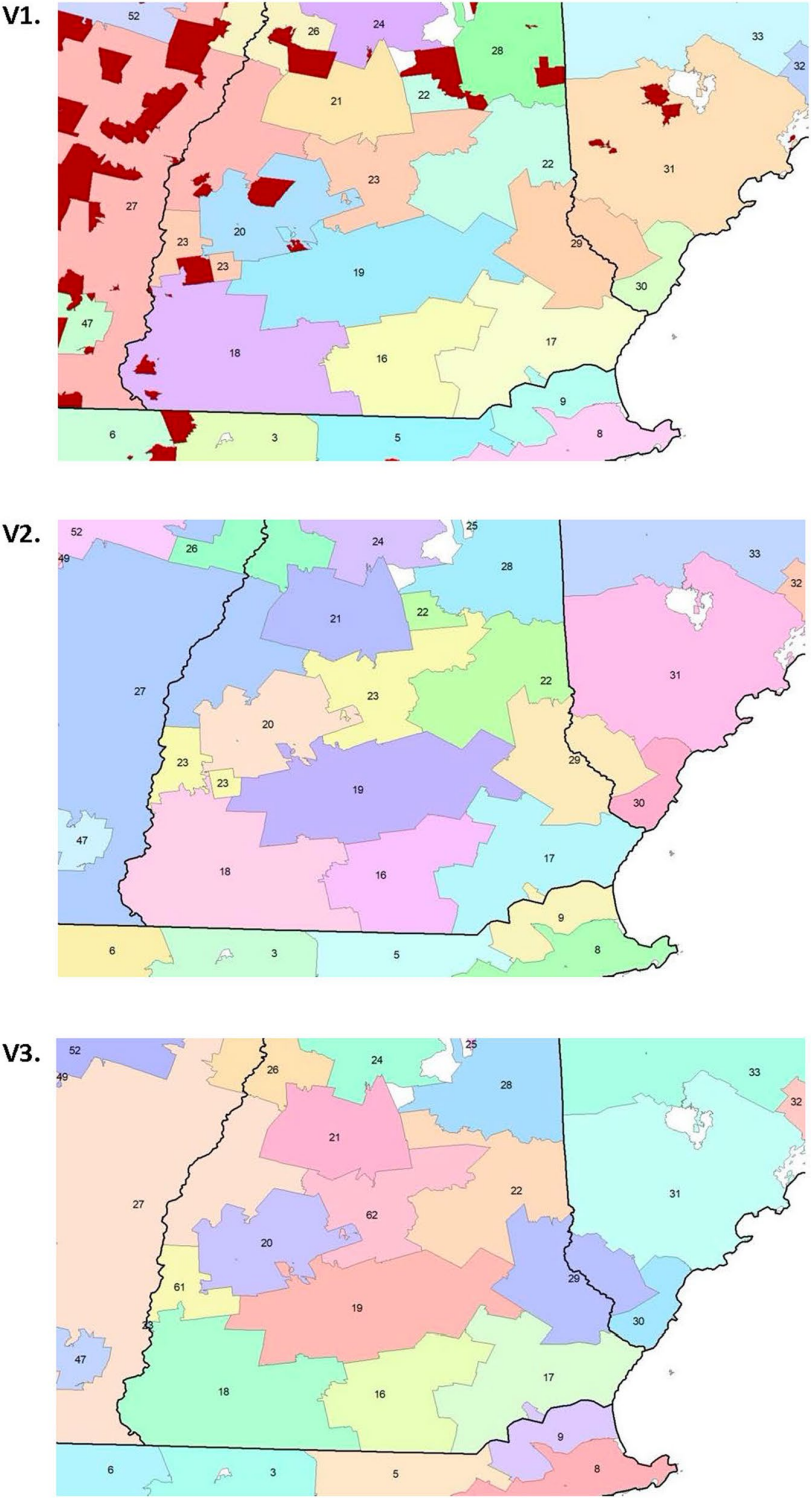
Maps describing an example of disjoint area resolution, southern New Hampshire, US, Three Versions (V1, V2,V3). Dark red indicates ZCTAs that were not included in initial clustering due to a low number of records. Gray represents areas that are not ZCTAs (e.g., lakes mountains, etc.). All other colors are used to differentiate boundaries. Version 1 (V1): Clusters 22 (upper middle) and 23 (middle left) have formed disjoints and there are unused ZCTAs. Version 2 (V2): Unused ZCTAs were added to nearby clusters, but disjoint issues with clusters 22 and 23 remain. Version 3 (V3): Disjoint resolution; cluster 22 was resolved by splitting a ZCTA off of cluster 23 to bridge the two disjoint pieces; cluster 23 was resolved by splitting the two pieces into new clusters 61 and 62, and a ZCTA from cluster 18 was used to bridge the gap between the two pieces of cluster 61

Once the clustering was completed, several additional steps needed to be taken. First, since clusters with low numbers of patients were not assigned in the initial clustering for stability reasons, they needed to now be assigned to clusters. Additionally, singleton clusters were considered an undesirable result, and they were treated as if they had not been assigned by the initial process. Finally, in some cases the clustering resulted in disjoint clusters, which are clusters in which some portion of the cluster is not contiguous with the rest. These disjoint areas arose primarily in rural areas with relatively low rates of PAC use. Resolution of these areas involved the evaluation and development of island and adjacency rules based on population characteristics and proximity rather than the clustering algorithm. These areas were assigned to the adjacent RSA with the highest population. Like other parts of our method, the assignment was an iterative process. We avoided reassignments that would create new disjoint areas. In addition, the process reassigned the smallest number of areas possible. As part of this process, we limited RSA populations to a minimum of 1,000 Medicare beneficiaries who resided in the area. We assigned these low population RSAs to the adjacent RSA with the highest population. Figure 2 illustrates the process of resolving disjoint areas in the southern portion of the US state of New Hampshire and is broken into three versions, i.e., V1, V2, and V3. In all versions, the color gray represents areas that are not ZCTAs (e.g., lakes mountains, etc.). In V1, dark red areas indicate ZCTAs that were not included in initial clustering due to a low number of records. In V2, unused ZCTAs were added to nearby clusters, but disjoints remain. In V3, disjoints were resolved by splitting and bridging clusters with a preference for using previously unassigned clusters. All other colors are used to differentiate boundaries.

### Area Characterization

We geographically clustered beneficiary ZCTAs with providers serving those ZCTAs. Our primary analysis involved the Ward’s clustering method and evaluation of cluster composition and stability with the Purity statistic by comparing clusters with and without home health services and by comparing 2013–14 clusters to 2015 clusters. Finally, we characterized RSAs descriptively by reporting the number of areas for each state, the percentage of those areas with IRF, SNF, and LTCH facilities for each state, select percentiles of state areas by the number of residents over 65 years of age, and the percentage of residents who traveled from the RSA where they live to a different RSA to receive care.

### Variables

RSA: Unique RSA variables were developed by aggregating clustered ZCTAs and assigning each group of ZCTAs a value from 1 to 1,711 nationally. Additionally, using ArcGIS, we constructed shape files for each RSA for mapping.

RSA Population: Using 2010 Census data, we obtained the number of adults over 65 by ZCTA. We aggregated ZCTA populations to RSAs using the RSA crosswalk file, then we calculated percentiles of the older adult population by RSA for each state.

RSA Travelers: A variable was created to reflect if the patient lived in the same RSA in which they received care. Patients were identified as travelers if they received care outside the RSA where they lived based on ZCTAs using the Medicare Beneficiary Summary files and ZCTA to RSA crosswalk file developed after the clustering approach described above. A binary variable, travelers, was created to show if the patient lived in the same RSA in which they received care. Traveling for post-acute rehabilitation has been shown to be significantly associated with the rehabilitative outcome successful community discharge [14]. Therefore, we account for this driver of geographic variation in PAC [14].

RSA Localization Index: RSAs developed from PAC admissions reflect the use of rehabilitation within small areas characterized by groups of ZCTAs. An important measure of the accuracy of small-area boundaries is the degree of localized utilization. To capture this key factor, we calculated a localization index for each RSA. The index is the proportion of beneficiaries residing in the same RSA where the rehabilitation care was provided [44] based on the grouping of ZCTAs within RSAs. We report the localization index as a function of IRF, SNF, and LTCH admissions only. Previously published research on small-area variation has utilized the preference index based on the location of the patient and provider [19]. However, in order to incorporate home health services that are not freestanding facilities like other PAC services, we modified this method and based it off of provider number and patient residence. In order to arrive at a modified localization index, we took 1 minus the proportion of travelers (those who traveled outside of their RSA of residence to receive care in another RSA):

Localization Index = 1 – (the proportion of travelers).

where the localization index is the percent of beneficiaries who received care in the RSA where they resided, and the proportion of travelers is the percent of beneficiaries who received care outside the RSA where they resided.

RSA Provider Makeup: A variable was created to show the types of PAC rehabilitation providers available for each RSA. This was done by linking the provider ZCTA to the RSA crosswalk file for IRF, SNF and LTCH providers to allow us to report the percentage of RSAs within a state who have IRF, SNF, and LTCH providers.

### RSA Stability

We evaluated the stability of RSAs in two ways using the Purity statistic [45]. The Purity statistic is a way to evaluate the quality of clusters. In this study, it shows the number of service areas that were clustered consistently. The larger the Purity statistic, the better the agreement [45]. First, we evaluated any potential impact of the inclusion of HHAs on RSA boundaries. To do this, we compared RSAs constructed from 2013 and 2014 of IRF, LTCH, and SNF admissions to RSAs constructed that included IRF, LTCH, SNF, and HHA. We calculated this Purity statistic. Second, we evaluated how stable 2013 and 2014 RSAs were compared to 2015 RSAs. We did this by comparing RSAs constructed from IRF, LTCH, SNF, and HHA admissions in 2013 and 2014 and compared them to RSAs constructed from 2015 data.

## Results

**Fig. 3:**
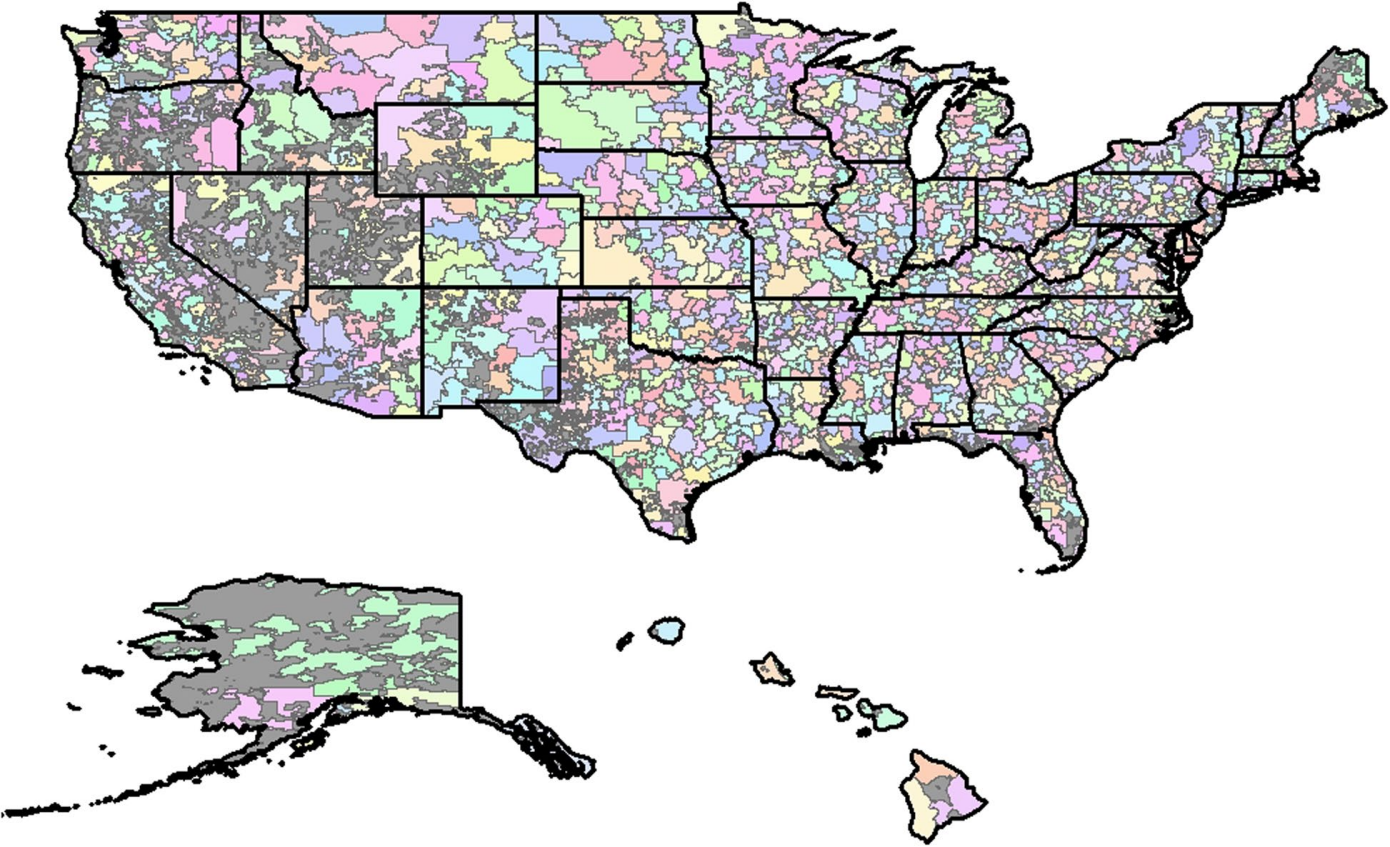
Map of Rehabilitation Service Areas for the entire United States. Gray represents areas that are not ZCTAs (e.g., lakes mountains, etc.). All other colors are used to differentiate boundaries

The clustering approach identified 1,711 unique RSAs distributed across the US (Fig. 3). Table 1 shows the distribution of RSAs per state, the percentage of IRF, LTCH, and SNF availability, and the localization index. The number of RSAs range from fewer than 10 per US state (Alaska, Connecticut, the District of Columbia, Delaware, Hawaii, Nevada, Rhode Island, and Wyoming) to over 70 RSAs (California, Illinois, North Carolina, Pennsylvania, and Texas). Similarly, the presence of IRF, LTCH, and SNF facilities varied across RSAs within each state. Nationally, 38.7% of RSAs had IRFs, 16.1% had LTCHs, and 99.7% had SNFs (Table 1). Traveling percentages also varied. Overall, the percentage of patients who traveled to a different RSA to receive care was 21.9%, with some states having RSAs where less than 15% of patients traveled, while others had RSAs where greater than 30% of residents who traveled. In different parts of the US, the propensity to travel varies as a function of differences of variables such as density of population and healthcare facilities, and the location of treatment facilities near RSA and state borders.

**Table 1:** Rehabilitation Service Areas (RSAs) by US state, percentage of RSAs with IRF, LTCH, and SNF availability, and the localization index

State Abbreviation—State Name	RSA Count	IRF Availability (%)	LTCH Availability (%)	SNF Availability (%)	LI (%)
Overall	1711	38.7	16.1	99.7	78.1
AK—Alaska	8	25.0	12.5	100.0	42.4
AL—Alabama	46	23.9	17.4	100.0	75.3
AR—Arkansas	49	42.9	12.2	100.0	74.4
AZ—Arizona	18	55.6	22.2	100.0	75.7
CA—California	81	43.2	16.0	98.8	76.8
CO—Colorado	28	25.0	10.7	96.4	77.7
CT—Connecticut	8	50.0	12.5	100.0	86.0
DC—District of Columbia	1	100.0	100.0	100.0	51.0
DE—Delaware	3	66.7	33.3	100.0	80.3
FL—Florida	51	56.9	31.4	100.0	81.1
GA—Georgia	50	40.0	20.0	100.0	69.1
HI—Hawaii	7	14.3	0.0	100.0	84.7
IA—Iowa	48	22.9	4.2	100.0	82.9
ID—Idaho	17	29.4	11.8	100.0	83.8
IL—Illinois	72	36.1	6.9	100.0	72.9
IN—Indiana	41	46.3	19.5	100.0	85.0
KS—Kansas	36	30.6	8.3	100.0	84.6
KY—Kentucky	49	32.7	10.2	100.0	76.9
LA—Louisiana	32	68.8	56.3	100.0	80.6
MA—Massachusetts	14	50.0	42.9	100.0	80.5
MD—Maryland	20	10.0	15.0	100.0	73.4
ME—Maine	14	35.7	0.0	100.0	80.6
MI—Michigan	56	42.9	25.0	100.0	76.8
MN—Minnesota	52	19.2	3.8	98.1	73.8
MO—Missouri	35	34.3	22.9	100.0	84.5
MS—Mississippi	38	28.9	18.4	100.0	75.6
MT—Montana	20	20.0	5.0	100.0	82.0
NC—North Carolina	71	28.2	11.3	98.6	75.2
ND—North Dakota	16	25.0	12.5	100.0	76.9
NE—Nebraska	33	21.2	6.1	100.0	84.4
NH—New Hampshire	14	28.6	0.0	100.0	71.8
NJ—New Jersey	11	72.7	54.5	100.0	78.0
NM—New Mexico	19	31.6	10.5	89.5	72.7
NV—Nevada	6	66.7	50.0	100.0	80.6
NY—New York	37	64.9	5.4	100.0	79.5
OH—Ohio	57	50.9	28.1	100.0	84.3
OK—Oklahoma	44	36.4	15.9	100.0	75.4
OR—Oregon	34	17.6	2.9	100.0	73.9
PA—Pennsylvania	77	61.0	18.2	100.0	73.5
RI—Rhode Island	2	50.0	0.0	100.0	86.4
SC—South Carolina	29	51.7	17.2	100.0	77.6
SD—South Dakota	19	21.1	5.3	100.0	80.3
TN—Tennessee	50	42.0	12.0	100.0	76.9
TX—Texas	104	50.0	34.6	100.0	77.7
UT—Utah	13	30.8	23.1	100.0	86.5
VA—Virginia	46	37.0	10.9	100.0	80.0
VT—Vermont	10	20.0	0.0	100.0	79.8
WA—Washington	45	37.8	4.4	100.0	74.3
WI—Wisconsin	46	32.6	6.5	100.0	80.4
WV—West Virginia	28	28.6	7.1	100.0	67.8
WY—Wyoming	6	50.0	16.7	100.0	76.3

*US* United States, *LI* Localization index, *RSA* Rehabilitation Service Area, *IRF* Inpatient rehabilitation facility, *LTCH* Long-term care hospital, *SNF* Skilled nursing facility

The median RSA population age 65 and older based on Census data ranged across states from 1,777 residents for an RSA in South Dakota to 40,227 residents in an RSA in Nevada (Table 2).

**Table 2:** Population percentiles across RSAs by US state (number of RSAs per state)

State Abbreviation (number of RSAs)—State	Minimum	10^th^ Percentile	Median	90^th^ Percentile	Maximum
Overall (1,711)	738	2,103	10,246	54,502	991,988
AK (8)—Alaska	915	915	5,546	21,112	21,112
AL (46)—Alabama	1,064	2,118	10,784.5	28,225	68,497
AR (49)—Arkansas	1,100	1,758	5,039	24,237	47,963
AZ (18)—Arizona	4,036	7,456	26,430	154,134	189,395
CA (81)—California	2,276	4,645	28,675	107,003	901,126
CO (28)—Colorado	882	1,366	6,309.5	65,187	143,471
CT (8)—Connecticut	11,040	11,040	24,407.5	198,171	198,171
DC (1)—District of Columbia	53,546	53,546	53,546	53,546	53,546
DE (3)—Delaware	33,426	33,426	34,666	61,359	61,359
FL (51)—Florida	1,907	12,379	49,840	115,492	286,942
GA (50)—Georgia	1,578	3,013.5	15,326.5	44,002.5	121,950
HI (7)—Hawaii	4,420	4,420	14,695	121,681	121,681
IA (48)—Iowa	988	1,485	3,886	26,980	57,369
ID (17)—Idaho	738	922	4,793	25,551	70,504
IL (72)—Illinois	775	2,043	9,162	42,485	423,305
IN (41)—Indiana	2,547	3,577	11,314	37,272	178,953
KS (36)—Kansas	1,082	1,168	3,603.5	28,276	88,470
KY (49)—Kentucky	1,054	2,190	5,563	28,163	88,949
LA (32)—Louisiana	1,185	3,337	9,829.5	38,444	63,806
MA (14)—Massachusetts	3,913	8,096	39,951	147,170	275,947
MD (20)—Maryland	5,660	6,335	19,886.5	104,212	167,026
ME (14)—Maine	2,162	2,918	9,453	33,426	51,520
MI (56)—Michigan	1,431	2,479	10,458.5	54,315	323,202
MN (52)—Minnesota	1,001	1,300	4,603	36,017	115,318
MO (35)—Missouri	1,652	2,469	10,640	41,040	247,173
MS (38)—Mississippi	859	1,527	5,728	27,842	55,699
MT (20)—Montana	1,136	1,199	2,993	20,448	21,601
NC (71)—North Carolina	1,979	4,316	11,372	38,588	81,956
ND (16)—North Dakota	1,090	1,442	3,698.5	19,510	20,282
NE (33)—Nebraska	849	1,077	2,939	10,414	85,521
NH (14)—New Hampshire	2,231	2,974	6,912.5	36,462	43,343
NJ (11)—New Jersey	14,538	16,436	69,893	251,249	271,533
NM (19)—New Mexico	2,208	2,303	6,974	27,308	111,200
NV (6)—Nevada	4,869	4,869	40,227	172,503	172,503
NY (37)—New York	4,720	5,501	25,876	151,384	991,988
OH (57)—Ohio	821	3,519	11,580	75,905	280,281
OK (44)—Oklahoma	935	1,404	6,499.5	17,387	112,184
OR (34)—Oregon	1,374	1,683	7,277	33,717	86,622
PA (77)—Pennsylvania	1,859	4,255	17,842	52,956	176,368
RI (2)—Rhode Island	20,011	20,011	75,943.5	131,876	131,876
SC (29)—South Carolina	2,253	4,579	14,394	43,754	79,266
SD (19)—South Dakota	973	987	1,777	27,152	31,029
TN (50)—Tennessee	1,035	2,971.5	10,082	44,153	85,821
TX (104)—Texas	803	1,553	13,076.5	61,220	226,240
UT (13)—Utah	1,447	1,539	8,914	42,780	98,610
VA (46)—Virginia	1,868	3,707	10,136	56,455	122,547
VT (10)—Vermont	3,201	4,020.5	6,235	22,218.5	25,740
WA (45)—Washington	820	1,602	11,334	48,304	78,808
WI (46)—Wisconsin	1,147	2,544	10,609	42,107	86,366
WV (28)—West Virginia	1,252	1,528	6,320	30,013	33,653
WY (6)—Wyoming	2,702	2,702	11,319	41,801	41,801

*RSA* Rehabilitation Service Area, *US* United States

RSA boundaries demonstrated stability in use in two ways. First, there was not a substantial impact on RSAs with the inclusion of HHA. We found a Purity statistic of 0.682 when we compared RSAs constructed from 2013 and 2014 data that included only IRF, LTCH, and SNF to RSAs constructed in that same period that included HHA admissions. Second, when evaluating the stability of the RSAs constructed from 2013 and 2014 data of IRF, LTCH, SNF, and HHA admissions to RSAs constructed from 2015 data, we found a similarly high Purity statistic of 0.806.

## Discussion

The purpose of this study was to define RSAs in the US and describe the methods employed to characterize national service areas for PAC in the US. We created and refined RSAs, and we determined RSA validity across the US. This report is the first national application to develop small areas for rehabilitation in the US. The resultant boundaries represent unique small areas for receipt of PAC services from four main provider types in the US: IRF, SNF, LTCH, and HHA.

The clustering approach identified 1,711 unique RSAs ranging from fewer than 10 to more than 70 per US state. We found variation in the availability of facilities across RSAs within each state; nationally, 38.7% of RSAs had IRFs, 16.1% had LTCHs, and 99.7% had SNFs. The RSA median population age 65 and older also varied across states with a range of 1,777 to 75,943 people. The localization index also varied. Overall, the percentage of patients who traveled to a different RSA to receive care was 21.9%, with a range of less than 15% to more than 30%.

Finally, our RSA boundaries were robust with a Purity statistic of 0.682 when comparing areas with and without home health services and 0.806 when comparing the clustering with 2012 and 2013 to 2015 data. This demonstrates good stability of these boundaries with the inclusion of home health services as well as over time when repeating the approach with later years of Medicare data.

RSA boundaries are necessary due to a lack of a comparable model that is appropriate for PAC services. The method we use in this study overcomes an issue in small-area variation research. Previous research has employed a preference index which creates a matrix that tabulates the ZIP code where the patient resides against the ZIP code of the provider of care [19]. This method is inappropriate for PAC. Home health care is a key part of PAC services that does not provide services out of freestanding facilities with an established ZIP code like other facilities in PAC such as IRFs, SNFs, and LTCHs [25]. Home health providers travel to a patient’s home address to provide care, therefore rendering the patient and provider ZIP code matrix inaccurate. To include home health care as providers in the construction of RSAs, we generated a matrix that tabulated patient ZIP code of residence against unique provider numbers. With this method, it did not matter where the HHA provided care, which thereby allowed our method to include HHAs appropriately.

As with other small-area variation boundaries in healthcare, RSAs need to be considered in light of some limitations. Most notably, RSAs are built based on data for older adults. Although Medicare beneficiaries represent the primary users of PAC rehabilitation services, and CMS drives reimbursement and policies for rehabilitation with other insurance providers, RSAs may not align with younger patients and those seen in other healthcare insurance systems. Additionally, RSAs were developed on patterns of IRF, SNF, LTCH, and HHA admissions and do not include other PAC providers also accessed by Medicare beneficiaries and other rehabilitation service recipients like outpatient services.

Finally, although our results report area-level descriptives in terms of population and availability, our boundaries are based on admissions only and do not take into account patient, facility, or contextual factors. Whereas previous research on geographic healthcare boundaries has used the preference index [19], we use a similar yet distinct concept—the localization index [44]. In Primary Care Service Area boundaries, the concept of the preference index implies patients may choose or prefer one provider over another. This assumption may not be appropriate for PAC services, for which clinical practice reflects that patients often are not able to choose their PAC provider. Therefore, in light of the methodological issues of the preference index discussed above, we abandoned the preference index in favor of focusing on patients who travel outside their RSA of residence to receive PAC services. This allows us to derive the localization index, i.e., one minus travelers. This is one way we evaluated the utility of RSAs. However, in this analysis, we did not evaluate the actual distance someone traveled for care, only whether they traveled outside of their RSA or not. This is in contrast to the method by Buntin et al. who, in 2010, used distance as an instrumental variable to describe PAC use [46]. Yet, our method is similar to that described by Dean et al. to describe PAC use across Hospital Service Areas [14].

RSAs allow us to look at transitions from acute care to PAC as a group. This is important as there are a variety of different admission policies, practices, and payment models which influence whether someone goes to one type of PAC vs another. Overall, the presence of one type of PAC provider influences the use of another provider type. RSAs allow us to look at the combined effect of PAC. This is an important piece to understanding a main driver of variation in healthcare use, access, quality, and cost. The ultimate goal of rehabilitation is to return home and engage in society [47, 48]. However, community participation is a broad term that encompasses many different definitions for researchers, policy makers, and providers. RSAs give us the capacity to describe and evaluate relationships between outcomes, community context, and provider availability and use. Without RSAs, geographic boundaries do not exist to evaluate these relationships appropriately. Measures such as successful community discharge [49, 50], a recent Medicare quality measure for PAC rehabilitation, require coordination among health care providers, social services agencies, community organizations, as well as family and caregiver support. Furthermore, these are influenced by social factors that impact the use of healthcare as well as behaviors and determinants that drive choices within the community. Social determinants of health are understudied drivers behind PAC access, utilization, outcomes, quality, and cost. RSAs give us an opportunity to examine these social factors.

## Conclusions

Health service researchers and policymakers confront many barriers to leveling quality up and cost down in practice. A critical step in understanding, addressing, and ultimately minimizing geographic differences in PAC outcomes is related to optimizing the geospatial area used to characterize geographic differences in PAC. We propose that current geographic boundaries may be insufficient to examine geographic variation in PAC. Current boundaries were not created for use in PAC and do not include characteristics unique to PAC use patterns. Therefore, we present our method that creates stable, RSA boundaries based on receipt of services across IRFs, LTCHs, SNFs, and HHAs in the US. We created RSAs for the entire US by grouping IRFs, SNFs, LTCHs, and HHA providers by clustering on the ZCTAs they serve, while considering distance between areas. RSAs provide policy makers, administrators, and researchers with a tool to characterize and understand drivers of variation in use, financing, quality, and outcomes of PAC.

## Data Availability

The data that support the findings of this study were used under a Data Use Agreement and are available from the Centers for Medicare and Medicaid Services through the Research Data Assistance Center. However, restrictions apply to the availability of these data, and therefore the data are not publicly available.

## Abbreviations

CMS: Centers for Medicare and Medicaid Services
HHA: Home health agency
IRF: Inpatient rehabilitation facility
LTCH: Long-term care hospital
PAC: Post-acute care
POS: Provider of service
RSA: Rehabilitation Service Area
SNF: Skilled nursing facility
US: United States
ZCTA: ZIP code tabulation area
ZIP: Zone improvement plan
